# Prevalence and antimicrobial susceptibility pattern of gram-negative bacteria contaminating the hands of patients’ visitors at regional referral hospitals in Dar-es-Salaam

**DOI:** 10.1371/journal.pone.0320700

**Published:** 2025-03-26

**Authors:** Ninael Jonas, Donath Mkenda Valerian, Stanslaus Henry, Emmanuel Magembe, Reuben Abednego, Loveness Urio, Eligius Lyamuya

**Affiliations:** 1 Department of Epidemiology and Biostatistics, Muhimbili University of Health and Allied Sciences, Dar es Salaam, United Republic of Tanzania,; 2 Tanzania Field Epidemiology and Laboratory Training Program, Dar es Salaam, United Republic of Tanzania,; 3 University of Dar es salaam, Dar es Salaam, United Republic of Tanzania,; 4 National Public Health Laboratory, Dar es Salaam, United Republic of Tanzania; Shiraz University of Medical Sciences, IRAN, ISLAMIC REPUBLIC OF

## Abstract

**Background:**

Hand contamination by bacteria is a significant source of infection transmission, especially in hospital settings. A healthcare-associated infection is one that a person contracts as a result of their interaction with a hospital. Health care workers’, patients’ and visitors’ hands are all transmission routes for infections, in particular bacterial infections. These infections elevate the economic burden on healthcare systems especially in low-income settings.

**Objective:**

To determine the prevalence of gram-negative bacteria hand contamination among patients’ visitors of referral hospitals in Dar es Salaam.

**Methodology:**

This was a cross-sectional study conducted at 3 regional referral hospitals: Amana, Temeke and Mwananyamala in March 2023. Dominant hand swabs from 388 systematically selected visitors were taken for bacterial culture and a short interview was conducted to assess factors associated with Gram-negative bacterial hand contamination. Hand swabs collected were cultured on Mac Conkey Agar (MCA), isolates were identified by VITEK MS and appropriate antibiotics were employed in antibiotic susceptibility testing of the isolated Gram-negative bacteria.

**Results:**

Prevalence of gram-negative bacterial contamination on visitors’ hands was 91 (21.1%) on entry and 103 (30.2%) on exit. The most common bacteria contamination was from *Klebsiella pneumoniae* on both entry and exit points, 37 (41%) and 57 (43%) respectively. Resistance to cephalosporins (Cefotaxime and Ceftriaxone) were the most pronounced. Proportion of ESBL-producing bacteria was significantly higher at exit than at entry. Bacterial contamination was associated with not washing hands APR = 1.5 (95% CI:1.03-2.17), offering services to the patient APR = 1.9 (95% CI:1.21-2.87) and longer hospital stays (more than 7 days) APR = 1.5 (95% CI:1.1-2.0).

**Conclusion:**

To prevent the transmission of bacteria, it is important to emphasize hand hygiene and exposure limits for visitors entering hospital environments.

## Introduction

Hand-transmitted infections have increasingly become a topic of public health importance. The emergence of COVID-19 had a global impact on people’s knowledge, perception and practice on hand hygiene. Hand contamination by pathogenic bacteria is a significant source of infection transmission, especially in hospital settings. Healthcare workers’, patients’ and visitors’ hands are all transmission routes for infections, particularly bacterial infections [1,2].

Hospitals are places where people get treatment, but they can also act as notorious infection reservoirs due to the influx and persistence of infectious agents within these facilities. If proper precautionary measures are not taken, these pathogens can easily be transmitted amongst its occupants[3]. Healthcare-associated infections (HCAIs) are infections occurring to patients in the process of receiving hospital care [2]. Studies show that infections that are acquired in hospitals by healthcare workers and patients’ visitors can also be considered as health care associated or nosocomial infections [4]. These infections are known to be more serious as they are often caused by antimicrobial resistant strains, causing longer hospital stay, higher treatment costs, and higher rates of morbidity and mortality.

Healthcare associated infections are prevalent both in developed and developing countries. In a comparative study between Argentina and England, the prevalence of HCAIs in Argentina was 11.3% and the most common were pneumonia, urinary tract infections (UTI), and surgical site infection [5]. In Singapore studies on HCAIs showed that prevalence was up to 11.9%, where the most common were clinical sepsis and pneumonia [6]. In Ibadan, Nigeria overall prevalence of infection was reported at 30.9% [7].

Multidrug resistant bacteria (MDR) bacteria is one of the key crises that the health system around the world faces presently [8]. They are known for causing high rates of morbidity and mortality globally as well as causing individuals, communities and countries great economic losses [9]. Several Gram-negative bacteria and *Staphylococcus* species pose a high risk to the health of human beings especially in the hospital setting [3,8]. In 2017, The World Health Organization (WHO) officially enumerated the antibiotic-resistant pathogens whereby Gram-negative bacteria were top on the list [10]. In the afore mentioned study in Nigeria, MDR bacterial infection prevalence was at 59.3%; MDR *Klebsiella* and MDR *Escherichia* species were reported as the most prevalent bacteria [7]. Furthermore, in Tanzania Majigo et al reported colonization with extended spectrum beta lactamase (ESBL) producing bacteria to be 59.7% with significant difference before and after hospitalization [11].

Healthcare associated infections pose a threat to the health system and to individuals, yet WHO reported that simple hand hygiene can quickly solve this problem and save lives [12]. Studies also report that hand hygiene is the prime means of preventing infection [13]. As pioneer, WHO created a guideline for health care workers (HCWs) on proper hand hygiene, called the five moments of hand hygiene [14]. Tanzania has adopted and established its own Infection Prevention Control (IPC) guidelines for HCWs and patients on how to conduct themselves and prevent further infection while at the health facility [15].

The practice of IPC during COVID-19 helped tremendously to reduce microbial infection [16]. This included the introduction of handwashing practices and use of sanitizers in many healthcare facilities. This was coupled with educating the public continuously on the need for hand hygiene especially when visiting patients in hospitals.

Most HCAIs which include MDR bacterial infections are reported to be brought by HCWs and patients [17] but there is a scarcity of information on the contribution of patients’ visitors to infection transmission. Some studies have shown that relatives who come to visit patients do not adhere to hand hygiene practices [18]. They could then be the transporters of pathogens from their homes to the hospitals and from the hospitals to their homes and communities.

This study aims to address the gap in knowledge regarding the prevalence of hand contamination among patients’ visitors. It will characterize the nature of this contamination and identify the factors that contribute to its occurrence.

## Methodology

### Ethical considerations

Ethical approval was obtained from the MUHAS Senate Research and Publications Committee, (Letter Ref: DA.282/298/01.C/1486). Permission to conduct the study was requested and obtained from the responsible administrative authorities in each hospital.

Participants were subjected to informed consent and the confidentiality of their identity was maintained by assigning them identification numbers instead of using names. Assent was obtained from parents/guardians for participants who were under-age (below 18 years). Further, access to databases was limited to the principal investigator and supervisors only.

### Study design and area

This was a cross-sectional study conducted at three regional referral hospitals in Dar es Salaam: Amana, Temeke, and Mwananyamala for a duration of one month, March 2023. Dar es Salaam was chosen for its large population, which increases infection transmission risk. The three hospitals, with bed capacities of 332 (Amana) and 370 each (Temeke and Mwananyamala), admit the majority of patients in the area. These hospitals follow Tanzania’s IPC guidelines, including hand hygiene practices and daily surface decontamination with disinfectants.

### Study population

The visitors of the inpatients at the three regional referral hospitals constituted the study population.

#### Inclusion and exclusion criteria.

Criteria for inclusion was visitors who came to see their patients in the evening during the data collection days.

Visitors who came to see their patients in the evening during the data collection days but did not enter the ward itself (some met with their patients in the corridors or other designated meeting area).

### Sample size estimation

The sample size was estimated using the Lwangwa and Lemeshow formula [19]. A prevalence of 50% of hand contamination was used in calculating the sample size due to paucity of published studies of the same kind. Confidence level considered was 95%, with a precision of degree of 5% and 10% nonresponse rate. Minimum sample size was estimated at 384. The sample size for each hospital was calculated using probability proportional to size, based on the hospital’s bed capacity, with the assumption that a higher number of beds correlates with more patients and, consequently, more visitors.

### Sampling method

Wards were purposively selected on the basis of studies that show more infections especially with MDR bacteria, are reported from them [20–22]. Wards selected were: medical, surgical, gynecology and pediatric wards. Amana RRH and Mwananyamala RRH had medical and surgical wards combined, furthermore, visitors were not allowed to enter the pediatrics and gynecology wards, this made it difficult to ascertain which actual wards the contamination was coming from. We decided to group wards into female medical and surgical ward and male medical and surgical ward.

Visitors to be swabbed and interviewed were selected using systematic random sampling at ward entry point on the days of data collection. A list of patients in the wards on each day was obtained and used to calculate the sampling interval. Data collectors selected every K^th^ person (expected number of visitors in the ward/required sample) on each day of sample collection, sample and data collection proceeded for multiple days until the allocated sample size for each hospital was reached.

### Sample collection and transportation methods

Hand swab specimen was collected from visitors’ hands as they entered the ward (sample A), and once again as they left the ward (sample B). Sterile swabs, moistened with 0.85% sterile normal saline were used to take sample. The swab was rolled around the palms, fingers, and in between fingers of the participant’s dominant hand. Two samples in each individual patient’s visitors were collected, one sample labelled A was collected when entering the hospital ward and sample B was collected from the same participant when leaving the hospital ward.

The swabs (A & B) were put in trypticase soy broth (TSB) and transported immediately to the National Public Health Laboratory (NPHL) in cooler box for testing.

### Laboratory processing

#### Bacterial culture and identification.

The samples were processed exclusively for Gram-negative bacteria because they constitute the majority of the WHO priority organism list. Also, financial constraints limited the feasibility of processing both Gram-negative and Gram-positive bacteria. Swabs were inoculated by striking on to MCA containing crystal violet. The inoculated MCA plates were incubated for 24 hours at 37 °C. The plates were then checked for bacterial growth and bacterial isolates from pure culture-positive plates were identified at the species level by their colony morphology. MCA cultures with mixed growth (53 original cultures) were sub cultured in BA, by picking the colonies that seemed different in morphology, until pure growth was achieved, then identification was done in VITEK MS; an automated mass spectrometry microbial identification system that uses Matrix Assisted Laser Desorption Ionization Time of Flight (MALD-TOF) technology systems [23].

#### Principle of VITEK MS.

The VITEK MS system is a MALDI-TOF (Matrix Assisted Laser Desorption Ionization – Time of flight). MALDI-TOF MS allows for the detection of high-abundance soluble proteins, including ribosomal and other structural proteins, directly from intact microbial cells resulting in spectra that are analyzed in the system database.

After growth on a culture medium a colony is picked using a Pick me pen and then smeared on the slide and matrix is added to it and left to dry for a few seconds. The slide is then introduced to a high vacuum environment and a precise laser blast ionizes the sample; proteins are released and accelerated by an electric charge, pass through right electrode. The proteins time of flight are recorded by mass, by charge then detected with the sensors to create a spectrum that represents the protein makeup of the specific bacteria.

#### Antimicrobial susceptibility testing.

AST was performed by using Kirby–Bauer disk diffusion method according to the Clinical and Laboratory Standards Institute (CLSI) M100 32^nd^ edition guideline [23]. The following antibiotics were set: For Enterobacteriaceae: Ceftriaxone (30µg), cefotaxime (30µg), ceftazidime (30µg), cefepime (30µg), Amoxicillin-clavulanic acid (20/10µg), amikacin (30µg), ampicillin (30µg), imipenem (10µg), ciprofloxacin (5µg), gentamicin (10µg). For Pseudomonas species: Ceftazidime (30µg), Cefepime (30µg), Imipenem (10µg), Gentamicin (10µg), Amikacin (30µg), and Ciprofloxacin (5µg). For Acinetobacter species: Ceftriaxone (30µg), cefotaxime (30µg), ceftazidime (30µg), cefepime (30µg), Amoxicillin-clavulanic acid (20/10µg), amikacin (30µg), imipenem (10µg), ciprofloxacin (5µg), gentamicin (10µg).

#### MDR bacteria.

MDR was defined as acquired non-susceptibility to at least one antibiotic in three or more antimicrobial classes [23]. Antimicrobial classes tested were Penicillin class (Ampicillin); Third-generation Cephalosporin class (Ceftazidime, Cefotaxime, Ceftriaxone); Fourth-generation Cephalosporins (Cefepime); Aminoglycosides class (Gentamycin, Amikacin); fluoroquinolones class (Ciprofloxacin); Carbapenems (Imipenem); Beta-lactam antibiotics (Amoxicillin-clavulanate).

#### ESBL screening procedure.

ESBL screening was done by using ceftazidime (30μg), and cefotaxime (30μg) during AST [24] and interpreted according to the CLSI guideline whereby those bacteria which resisted these drugs were considered as ESBL producers and thus subjected to ESBL production phenotypic confirmation test. Double Disk Synergy Test (DDST) was used as a phenotypic confirmation test whereby a suspension of microorganisms was prepared and equilibrated to match 0.5 McFarland’s standard, then a lawn culture on MHA was prepared then ceftazidime (30 μg) and cefotaxime (30 μg) were placed at a distance of 20 mm each around augmentin disc (20 μg amoxicillin +  10 μg clavulanic acid) and incubated at 37 ºC overnight. The organisms were phenotypically confirmed to be ESBL-producers when the zone of inhibition around any of these third cephalosporin discs showed a clear-cut increase towards augmentin disc.

#### Quality control.

It was by using standard organisms American Type Culture Collection (ATCC) control strains for *Klebsiella pneumoniae* ATCC 700603 and *Escherichia coli* ATCC 25922 for control in AST procedure and *Klebsiella pneumoniae* ATCC 700603 for control in ESBL screening procedure were used as control organisms.

Control for media used was done by first performing sterility check of new batch of culture media; two plates of new batch of culture media was incubated for 24 hours at 37°C and observed for any growth. Then a performance check was done whereby two plates of MCA were inoculated with known isolates of *Escherichia coli* and *Pseudomonas aeruginosa* in order to observe for lactose fermentation.

#### Storage of the isolated bacteria from visitors’ hand swab samples.

The bacteria isolated from the visitors’ culture positive hand swab samples were then stored in cryovial tubes containing storage media solution at -80◦C temperature. The isolates stored can be used for further studies like molecular characterization of the species and mechanisms of drug resistance.

#### Interview with participants.

When exiting the ward, the patient’s visitor was interviewed to assess some of the practices while at the hospital and ward. The patient’s visitor was queried on sociodemographic factors including age, address, sex, occupation and education level. They were asked of their hospital practices including whether they washed their hands on entry and at exiting the ward, whether they offered the patient any services, touched them or sat down in the ward.

### Statistical analysis

Data were collected using Open Data Kit (ODK) and analyzed with Stata version 15.0 (Stata Corporation, College Station, TX). Sociodemographic characteristics were summarized using frequencies and proportions, and descriptive statistics presented hospital practices. The prevalence of bacterial contamination on visitors’ hands was determined from positive laboratory cultures, with common pathogenic bacteria identified by their proportions. Bacterial contamination proportions were analyzed across sociodemographic and hospital practice characteristics, and a chi-square test assessed whether there is significant difference between independent variables on the outcome

A Modified Poisson regression model was used to identify factors associated with bacterial hand contamination among visitors. Prevalence ratios and corresponding 95% confidence intervals were reported. Akaike’s information Criteria (AIC) was used to determine the best model in multivariate analysis [25].

## Results

### Sociodemographic characteristics of study participants

**Fig 1:**
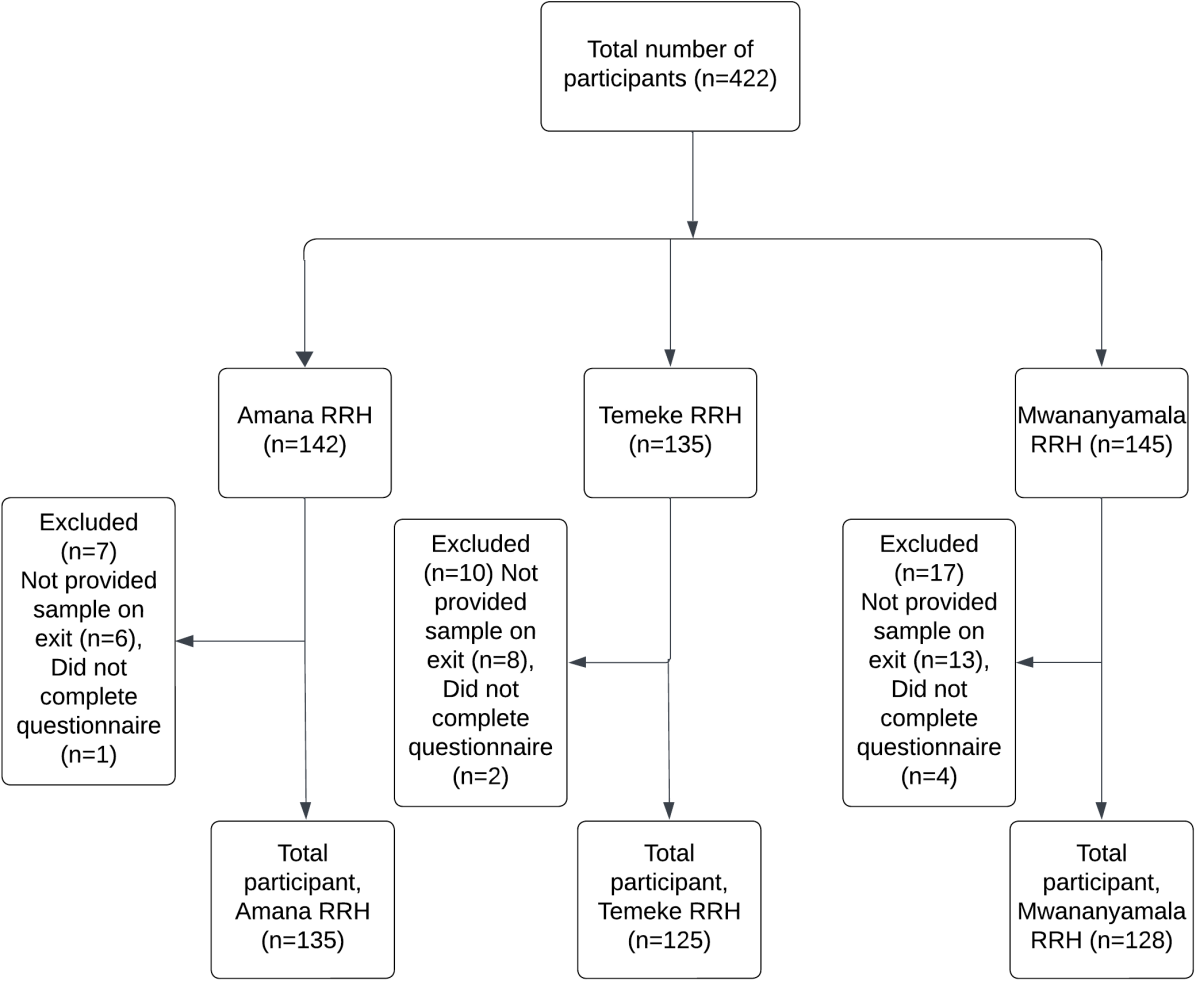
Study flow chart.

Of the 422 patient visitors, 388 (91.9%) consented and participated in the study (Fig 1). The majority of participants were from Amana RRH (34.8%), The median age of the participants was 38 years (IQR:36.9-39.4). Most of the visitors were female (70.4%) with majority of them from Ilala (29.6%) and Temeke (26.5%). More than half of the participant had primary level education or lower (57.7%) and were self-employed (54.1%) (Table 1).

**Table 1:** Sociodemographic characteristics of study participants N = 388.

Variable	n (%)
**Hospital**	
Amana RRH	135(34.8)
Mwananyamala RRH	128(33)
Temeke RRH	125(32.2)
**Age categories (in years)**	
<=18	5(1.3)
19-35	183(47.2)
36-64	189(48.7)
65 +	11(2.8)
**Sex**	
Female	273(70.4)
Male	115(29.6)
**Address**	
Ilala	115(29.6)
Kigamboni	12(3.1)
Kinondoni	86(22.2)
Temeke	103(26.5)
Ubungo	53(13.7)
Outside Dar es Salaam	19(4.9)
**Education level**	
Primary or lower	224(57.7)
Secondary or above	164(42.3)
**Occupation status**	
Unemployed	121(31.2)
Self employed	210(54.1)
Formally employed	57(14.7)

### Participants practice while at the hospital

About half of the visitors went to the female medical/surgical wards (49.0%). Most visited patients who had been admitted for 1-2 days (77.7%). The majority of visitors reported visiting only once (84.0%) and not touching or shaking hands with the patient (59.7%). Most also reported not taking a seat while in the ward (72.4%) or offering services such as feeding, turning, or changing clothes for the patient (85.8%). More than half of the participants reported not washing their hands upon entering (62.0%) or exiting the ward (71.6%) (Table 2).

**Table 2:** Hospital practice characteristics of participants (N = 388).

Variable	n (%)
**Ward admitted**	
Female medical/surgical	190(49)
Male medical/surgical	149(38.4)
Other^1^	49(12.6)
Total	388(100)
**Duration of admission (days)**	
<7	271(77.7)
≥ 7	78(22.3)
Total^*^	349(100)
**Number of Visits to patient**	
Multiple	62(16)
Once	325(84)
Total^*^	387(100)
**Handshake with patient**	
No	231(59.7)
Yes	156(40.3)
Total^*^	387(100)
**Visitor sat in ward**	
Not seated	280(72.4)
Seated	107(27.6)
Total^*^	387(100)
**Visitor offered services to patient**	
No	333(85.8)
Yes	55(14.2)
Total	388(100)
**Visitor washed hands on entry**	
No	240(62)
Yes	147(38)
Total^*^	387(100)
**Visitor washed hands on exit**	
No	277(71.6)
Yes	110(28.4)
Total^*^	387(100)

^1^Other included Gynecology and Pediatric wards.

* Variable totals differ due to non-response in some of the questions.

### Prevalence of Gram-negative bacteria contaminating the hands of patients’ visitors

**Fig 2:**
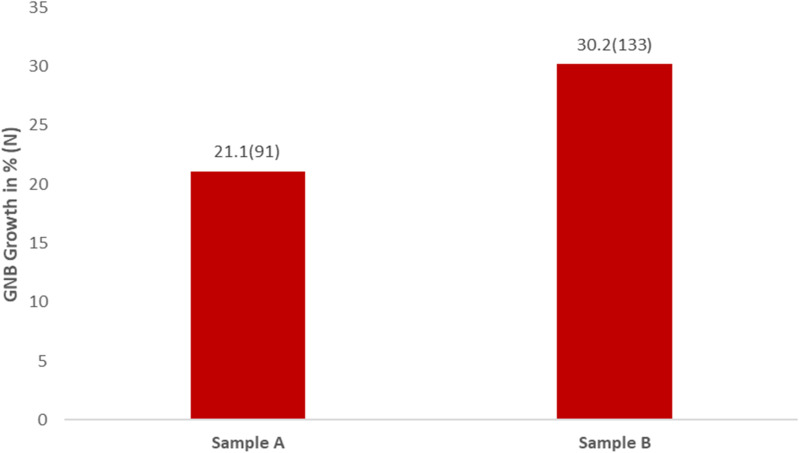
Proportion of bacterial growth in sample A and B.

The study found that the prevalence of Gram-negative bacterial contamination on the hands of patients’ visitors was 21.1% upon entering and 30.2% upon exiting the hospital wards (Fig 2).

**Fig 3:**
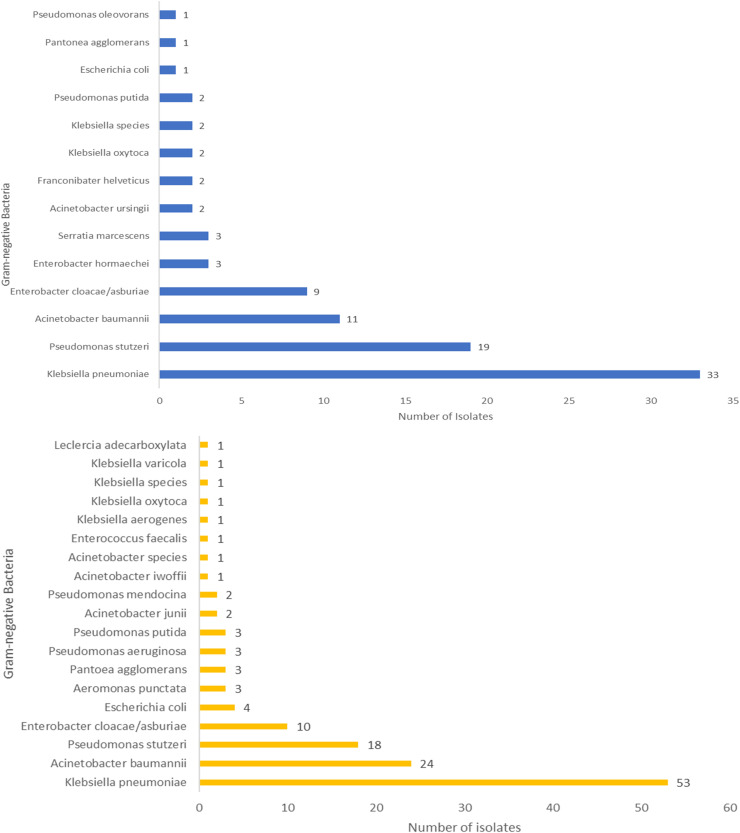
Gram-negative bacteria isolated from visitors in sample A and B.

Laboratory analysis of hand swab samples from visitors, designated as sample A (n = 91) and sample B (n = 133), revealed that *Klebsiella pneumoniae* was the most prevalent bacteria in both entry (41.0%) and exit (43.0%) samples (Fig 3_Sample A, Fig 3_Sample B). A total of 53 samples contained mixed isolates; 115 subcultures were performed, yielding 77 isolates.

### Comparison of proportions of Gram-negative bacteria on participants’ hands between entry and exit

Analysis showed that 19.3% of the patients’ visitors came in with no Gram-negative bacterial growth in their hands and left with at least one Gram-negative bacteria contaminating their hand. Conversely, 10.8% of visitors entered with Gram-negative bacteria but did not leave with it. The difference between proportion of visitors who left with GNB having not entered with them to those who left with GNB having entered with them was significant (Table 3). It is important to note that the difference between proportion of visitors who left with GNB having not entered with them“was investigated at species level and not indicating any genetic linkages.

**Table 3:** Proportions of Gram-negative bacteria (GNB) contaminating the hands of patients’ visitors at entry and exit N = 388.

Scenarios	Number	Proportion (%)	P value
Left with GNB but did not enter with GNB	75	19.3	0.0009^a^
Left with GNB and entered with GNB	42	10.8	0.0009^b^
Entered with GNB but did not leave with it	40	10.3	0.8153^c^
Entered with GNB and left with GNB	42	10.8	0.8153^d^

a, b, c, d – Difference in proportion test

### Proportion of gram-negative growth against socio-demographic characteristics

There was no significant contribution of the sociodemographic factors to having a positive gram-negative bacterial growth on entry or exit (see Table 4).

**Table 4:** Proportion of Gram-negative growth on patients’ hands upon entry and exit against socio-demographics.

Variable	Entry		*p-value* ^1^	Exit		*p-value* ^1^
	Growth (%)	Total		Growth(%)	Total	
**Hospital**						
Amana RRH	21(15.6)	135	0.065	40(29.6)	135	0.953
Mwananyamala RRH	35(27.3)	128		38(29.7)	128	
Temeke RRH	26(20.8)	125		39(31.2)	125	
**Age**						
≤18	2(40.0)	5	0.352^a^	1(20.0)	5	0.992^a^
19-35	43(23.5)	183		55(30.1)	183	
36-64	36(19.0)	189		58(30.7)	189	
≥64	1(9.1)	11		3(27.3)	11	
**Sex**						
Female	59(21.6)	273	0.723	87(31.9)	273	0.258
Male	23(20.0)	115		30(26.1)	115	
**Address**						
Ilala	23(20.0)	115	0.136^a^	36(31.3)	115	0.881^a^
Kigamboni	5(41.7)	12		3(25.0)	12	
Kinondoni	24(27.9)	86		26(30.2)	86	
Temeke	16(15.5)	103		29(28.2)	103	
Ubungo	9(17.0)	53		15(28.3)	53	
Outside Dar	5(26.3)	19		8(42.1)	19	
**Education**						
Primary and below	42(18.8)	224	0.18	70(31.3)	224	0.583
Secondary and above	40(24.4)	164		47(28.7)	164	
**Occupation**						
Unemployed	29(24)	121	0.651	39(32.2)	121	0.817
Self-employed	42(20)	210		62(29.5)	210	
Formally employed	11(19.3)	57		16(28.1)	57	

^1^A chi square association test was used to check for association between sociodemographic factors and presence of growth of bacteria in Sample A.

^a^Fisher’s exact test of association

### Proportion of gram-negative bacterial growth against participant practice while at the hospital

Analysis showed that the exposures duration of admission, offering a handshake or touch, seating, offering services and not washing hands on entrance had a significant contribution to isolating Gram-negative bacteria in sample B (on exit) as shown in Table 5.

**Table 5:** Proportion of gram-negative growth in Sample B against practice while at the hospital.

Variable	Gram Negative Bacterial growth		*p*-value^1^
	Growth (%)	Total	
**Ward relative is admitted**			
Female medical/surgical	56(29.5)	190	0.559
Male medical/surgical	43(28.9)	149	
Others	18(36.7)	49	
**Duration of admission (days)**			
<7	72(26.6)	271	0.015
≥ 7	32(41.0)	78	
**Number of Visits**			
Multiple	58(25.1)	62	0.060
Once	59(37.8)	325	
**Visitor handshake with patient**			
No	58(25.1)	231	0.008
Yes	59(37.8)	156	
**Visitor sat in ward**			
Not seated	75(26.8)	280	0.018
Seated	42(39.3)	107	
**Visitor provided services**			
No	88(26.4)	333	0.000
Yes	29(52.7)	55	
**Visitor washed hands on entrance**			
No	63(26.3)	240	0.030
Yes	54(36.7)	147	
**Visitor washed hands on exit**			
No	88(31.8)	277	0.298
Yes	29(26.4)	110	

^1^A chi square association test was used to check for association between visitors’ practice factors and presence of growth of bacteria in Sample B

### AST pattern of gram-negative bacteria contaminating hands of visitors

#### Sample A.

Acinetobacter species showed resistance to cefotaxime and ceftriaxone by 84.6% and 69.2% respectively with less resistance to cefepime and ceftazidime 38.5% and 30.8%, respectively. Enterobacter species showed resistance to cefotaxime and ceftazidime by 50.0%, 41.7%, respectively and Augumentin and Ampicilin by 83.3% each. *Klebsiella* species showed resistance to Ampicilin, cefotaxime, Augumentin and Ciprofloxacin by 94.6%, 70.3%, 67.6% and 54.1%, respectively (Table 6).

**Table 6:** Antimicrobial resistance pattern of Gram-negative bacteria in sample A and B contaminating hands of patients’ visitors’.

	Total	AUG	AMP	FEP	CAZ	IMI	CN	AK	CIP	CTX	CRO
**Sample A**											
*Acinetobacter species*	13	NA	NA	5(38.5)	4(30.8)	1(7.7)	1(7.7)	0(0)	0(0)	11(84.6)	9(69.2)
*Enterobacter species*	12	10(83.3)	10(83.3)	2(16.7)	5(41.7)	0(0)	2(16.7)	2(16.7)	2(16.7)	6(50)	2(16.7)
*Klebsiella species*	37	25(67.6)	35(94.6)	14(37.8)	17(45.9)	4(10.8)	6(16.2)	8(21.6)	20(54.1)	26(70.3)	13(35.1)
*Pseudomonas species*	22	NA	NA	0(0)	0(0)	0(0)	1(4.5)	1(4.5)	0(0)	NA	NA
Other bacteria^1^	7	2(28.6)	7(100)	0(0)	1(14.3)	0(0)	0(0)	0(0)	0(0)	3(42.9)	5(71.4)
**Sample B**											
*Acinetobacter species*	28	NA	NA	3(10.7)	8(28.6)	1(3.6)	1(3.6)	1(3.6)	2(7.1)	24(85.7)	21(75)
*Enterobacter species*	10	9(90)	8(80)	4(40)	4(40)	1(10)	3(30)	1(10)	3(30)	5(50)	4(40)
*Klebsiella species*	57	42(73.7)	52(91.2)	31(54.4)	34(59.6)	13(22.8)	12(21.1)	9(15.8)	34(59.6)	44(77.2)	29(50.9)
*Pseudomonas species*	26	NA	NA	0(0)	0(0)	0(0)	0(0)	0(0)	0(0)	NA	NA
Other bacteria^2^	12	4(33.3)	8(66.7)	0(0)	1(8.3)	0(0)	0(0)	1(8.3)	3(25)	5(41.7)	1(8.3)

^1^Other bacteria included *Escherichia coli* (1), *Franconibater helveticus* (2), *Pantonea agglomerans* (1), *Serratia marcescens* (3).

^2^Other bacteria included *Aeromonas punctata* (3), *Escherichia coli* (4), *Leclercia adecarboxylata* (1), *Pantonea agglomerans* (3), *Enterococcus faecalis* (1)NA- Not applicable

AUG, Augumentin; AMP, Ampicilin; FEP, Cefepime; CAZ, Ceftazidime; IMI, Imipinem; CN, Gentamicin; AK, Amikacin; CIP, Ciprofloxacin; CTX, Ceftaxime; CRO, Ceftriaxone.

#### Sample B.

*Acinetobacter* species isolated showed resistance to Cefotaxime and Ceftriaxone by 85.7% and 75.0%, respectively. *Enterobacter* species showed resistance to Augumentin, Ampicillin and Cefotaxime by 90.0%, 80.0%, 50.0%, respectively. *Klebsiella* species showed resistance to Ampicilin, Cefotaxime, Augumentin by 91.2%, 77.2%, 73.7%, respectively (Table 6). Additionally, susceptibility tables for samples A and B are in supporting information S1_Table.

### MDR and ESBL producers

**Fig 4:**
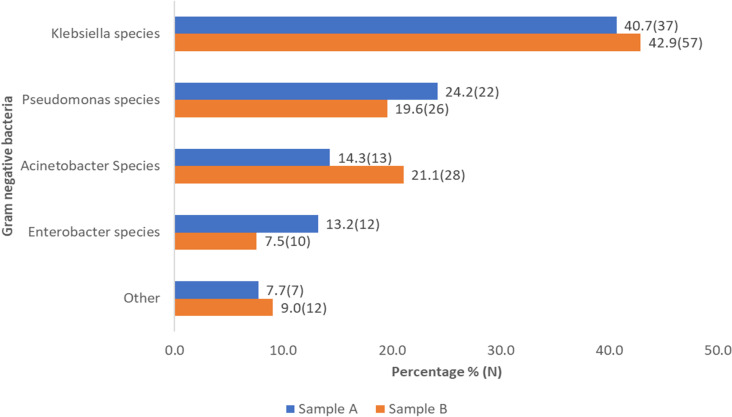
Proportion of MDR and ESBL producing Gram-negative bacteria isolated from participants’ hands.

In sample A, 36.3% of isolates and in sample B 30.9% of isolates were resistant to three or more classes of antibiotics, that is, were multi drug resistant (MDR) (Fig 4). The overall proportion of ESBL producers in this study was 18.1%. the proportion of ESBL for isolates from sample A was 14.3% and from sample B was 24.3%. the majority of ESBL producer from both samples were *Klebsiella pneumoniae.* Proportion test showed no significant difference between MDR in sample A and B, the test showed significance between ESBL in sample A and B (Table 7).

**Table 7:** Proportion test results on MDR and ESBL for sample A and B.

	Sample categories	Frequencies	Proportions	p value^1^
MDR	Sample A	33	0.36	0.398
	Sample B	41	0.31	
ESBL	Sample A	13	0.14	0.033
	Sample B	32	0.24	

^1^A proportion test was used to check for difference in MDR and ESBL proportions between Sample A and Sample B

### Factors associated with gram-negative bacterial growth

The independent variables were tested against the positive Gram-negative bacterial growth in both samples A and B to determine the associations, bivariate and multivariate modals were run. There was a significant association between Gram-negative bacterial growth in samples A and Mwananyamala RRH but no other significant associations with other sociodemographic variables (age, sex, education, occupation, ward visited). (Table 8 and 9).

**Table 8:** Factors associated with gram negative bacterial growth in sample-A study participants’ hands.

Variable	Total	%	Bivariate PR (95%CI)	*p*-value	Multivariate APR (95% CI)	*p*-value
**Hospital**						
Amana RRH	135	34.8	**Ref**		**Ref**	
Mwananyamala RRH	128	33.0	1.76 (1.08-2.85)	0.023	1.66 (1.01-2.72)	0.045
Temeke RRH	125	32.2	1.34 (0.79-2.25)	0.275	1.43 (0.84-2.44)	0.191
Total	388					
**Age**						
<=18	5	1.3	**Ref**		**Ref**	
19-35	183	47.2	0.59 (0.19-1.78)	0.346	0.56 (0.17-1.84)	0.335
36-64	189	48.7	0.48 (0.16-1.45)	0.192	0.52 (0.16-1.72)	0.286
64 +	11	2.8	0.23 (0.03-1.97)	0.178	0.23 (0.03-2.10)	0.194
Total	388	1.3				
**Sex**						
Female	115	29.6	**Ref**			
Male	273	70.4	0.93 (0.60-1.42)	0.724		
Total	388					
**Education**						
Primary/below	164	42,3	0.77 (0.52-1.13)	0.179	0.86 (0.59-1.25)	0.418
Secondary/upper	224	57.7	**Ref**		**Ref**	
Total	388					
**Occupation**						
Unemployed	57	14.7	1.24 (0.67-2.31)	0.493		
Self employed	121	31.2	1.04 (0.57-1.88)	0.907		
Formally employed	210	54.1	**Ref**			
Total	388					
**Ward visited**						
Female medical/surgical	190	49.0	**Ref**		**Ref**	
Male medical/surgical	149	38.4	0.74 (0.49-1.13)	0.162	0.81 (0.53-1.23)	0.313
Other	49	12.6	0.48 (0.22-1.07)	0.072	0.53 (0.24-1.15)	0.109
Total	388					

**Table 9:** Factors associated with Gram-negative bacterial growth in sample B study participants’ hands.

Variable	Total	%	Bivariate PR (95%CI)	*p*-value	Multivariate APR (95% CI)	*p*-value
**Hospital**						
Amana RRH	135	34.8	**Ref**			
Mwananyamala RRH	128	33.0	1.00 (0.69-1.45)	0.992		
Temeke RRH	125	32.2	1.05 (0.73-1,52)	0.783		
Total	388					
**Age**						
<=18	5	1.3	**Ref**			
19-35	183	47.2	1.50 (0.26-8.82)	0.652		
36-64	189	48.7	1.53 (0.26-8.99)	0.635		
64 +	11	2.8	1.36 (0.18-10.11)	0.762		
Total	388					
**Sex**						
Male	115	29.6	**Ref**			
Female	273	70.4	1.22 (0.86-1.74)	0.267		
Total	388					
**Education**						
Secondary/upper	164	42,3	**Ref**			
Primary/lower	224	57.7	1.09 (0.80-1.49)	0.585		
Total	388					
**Occupation**						
Formally employed	57	14.7	**Ref**			
Unemployed	121	31.2	1.15 (0.70-1.87)	0.580		
Self employed	210	54.1	1.05 (0.66-1.68)	0.832		
Total	388					
**Ward**						
Female medical/surgical	190	49.0	**Ref**			
Male medical/surgical	149	38.4	0.98 (0.70-1.37)	0.903		
Other	49	12.6	1.25 (0.81-1.91)	0.314		
Total	388					
**Duration (days)**						
<7	271	77.7	**Ref**		Ref	
7 +	78	22.4	1.54 (1.11-2.15)	**0.010**	1.50 (1.1-2.0)	0.012
Total	349					
**Number of visits**						
Once	325	84.0	**Ref**			
Multiple	62	16.0	1.42 (1.00-2.02)	0.047		
Total	387					
**Visitor handshake with patient**						
No	231	59.7	**Ref**		**Ref**	
Yes	156	40.3	1.51 (1.12-2.03)	0.008	1.3 (0.9-2.0)	0.175
Total	387					
**Visitor sat in ward**						
Not seated	280	72.4	**Ref**		**Ref**	
Seated	107	27.7	1.47 (1.08-1.99)	0.014	0.9 (0.54-1.40)	0.78
Total	387					
**Visitor provided services to patient**						
No	333	85.8	**Ref**		**Ref**	
Yes	55	14.2	2.00 (1.47-2.72)	0.000	1.9 (1.21-2.87)	0.005
Total	388					
**Visitor washed hands on entrance**						
No	240	62.0	0.71 (0.53-0.96)	0.028	0.66 (0.47-0.93)	0.018
Yes	147	38.0	**Ref**		**Ref**	
Total	387					
**Visitor washed hands on exit**						
No	277	71.6	1.21 (0.84-1.72)	0.306	1.5 (1.03-2.17)	0.032
Yes	110	28.4	**Ref**		**Ref**	
Total	387					

There was significant association between Gram-negative bacterial growth in sample B with duration of stay in the hospital, offering services to the patient, not washing hands on entry and exit. Those visitors whose patients had been admitted longer than 7 days had 1.5 the prevalence of having Gram-negative contamination on their dominant hand than those admitted less than 7 days, APR = 1.5(95% CI:1.05-2.12). Visitors who reported to offer services to the patient had almost twice the prevalence of Gram-negative bacterial contamination on their dominant hand as compared to those who did not offer their patients any services, APR = 1.9 (95% CI:1.21-2.87). Visitors who reported not to have washed their hands on exit had 1.5 the prevalence of having Gram-negative bacterial growth on their dominant hand at exit than those who reported to have washed their hands at exit, APR = 1.5 (95% CI:1.02-2.19).

Those who reported not to have washed their hands at the entrance of the ward had 34% less prevalence of the outcome than those who reported to have washed their hands at the entrance of the ward, APR = 0.66 (95% CI:0.47-0.93).

## Discussion

This study demonstrated that the prevalence of Gram-negative bacterial contamination on patients’ visitors’ hands was 21.1% on entry and 30.2% on exiting the hospital. This indicates that visitors not only bring a significant number of pathogenic bacteria when they enter the ward but importantly, they also take pathogenic bacteria back into the community when they leave the hospital. Due to paucity of data on prevalence of visitors’ hands contamination with bacteria, the findings of this study have been compared to studies on HCWs and patients.

This prevalence is similar to the reported prevalence of bacterial contamination of hands among HCWs in Mwanza (26.4%) [26], in Brazil (37.8%) [27], and Iran (39.3%) [28]. The prevalence observed in this study differs from the prevalence of Gram-negative pathogens isolated from neonate mother’s hands at Bugando Medical Center (18.5%) [29], this could be because mothers of neonates are subjected to and are more conscious of taking precautions before they handle their babies than other visitors with their adult patients. The prevalence in this study also differs from that reported from Bugando Medical Center from samples taken from June 2013 to May 2015 which was 13.2% [30]. This could be because these samples were not from visitors’ hands, but could indicate an increase in Gram-negative bacteria colonization at hospitals. The prevalence also differs from that reported of tertiary hospital health care workers in Greece (45.6%) [31] and on patient’s hands contamination in Iran (39.3%) [28]. This disparity could indicate that the magnitude of contamination is higher among HCWs and patients. But it could also be due to differences in wards where the HCWs were sampled from, in this study the visitors were selected mainly from medical and surgical wards. Nonetheless, the prevalence of contamination observed indicates the contribution of visitors to the pathogen pool in wards they visit and the magnitude of bacteria they possibly take home after visiting these hospitals.

The most common isolated bacterial species in this study was *Klebsiella pneumoniae* in both entry and exit samples (41% and 43%, respectively). This finding is similar to those reported in several other studies on bacteria isolated from hands of HCW and patients where Klebsiella species have always shown prominence [31,32]. *Klebsiella pneumoniae* is responsible for many blood stream infections and is known to be resistant in many cases to several classes of antimicrobial drugs. Klebsiella species are also known to be commonly transmitted through individuals’ skin or shared items and further worsened by not washing hands after offering services to a patient [32] which is a factor associated with this contamination.

The isolated bacteria in the entry and exit sample showed similar resistance pattern to antibiotics: penicillins and cephalosporins. Few studies have assessed the antibiotic resistance pattern of isolates from relatives’ hands; however, we may compare our results with those done on hospital surfaces and from clinical samples. Similar resistance patterns have been reported in a recent study on contaminated surfaces at tertiary hospitals in Dar es Salaam where highest resistances were observed to Ceftriaxone (63-100%) [33]. Other studies in Tanzania and Zimbabwe have also reported an increase of resistance of Gram-negative bacteria in clinical samples to third-generation cephalosporins over recent years [34–36] by Klebsiella and Acinetobacter species. The demonstration of Klebsiella species resistance to cephalosporins, augumentin and ampicillin is not new [34]. This depicts the difficulty in treating this kind of infection, leading to longer hospital stays, increased economic burden and even increased mortality.

The proportion of MDR bacteria found in this study were 36.3% at entrance and 30.8% at exit. This finding is different from that reported of MDR bacteria causing surgical site infection at Muhimbili National Hospital 61.4% [35], and MDR reported in an ICU in a study done in Uganda 58% [36] and in Zimbabwe (75%) on close contact surfaces and health care workers [37]. This disparity could be due to differences in sampled ward: The Zimbabwe study was conducted in ICU, and among HCWs who are more likely to be contaminated as they have more contact time with patients. The proportion of ESBL producers in this study was 18.1% with majority being *Klebsiella pneumoniae*. This finding is similar to that reported from a study done on skilled care residents where ESBL Klebsiella were 18.0% [38] and 18.6% for those in ICU. Apparently, these findings suggest that visitors to hospitals contribute significantly to the MDR bacteria circulating in the hospitals and by deduction, that circulating in the community.

The proportion of MDR at entrance of ward was higher than that at exit of ward in this study. This is different from what is expected since the hospital is known to be a reservoir of infection [3]. A proportion test showed that this difference was not statistically significant, but also indicates that this is a vicious cycle since the visitors seem to be carrying around the same proportion of MDR. On the other hand, proportion of ESBL producing Gram-negative bacteria was significantly higher among those exiting than those entering the hospital supporting the above-mentioned theory. This supports the hypothesis that visitors carry significant proportions of resistant bacterial strains back in to the community and later into the hospital, creating a vicious cycle of MDR bacterial infections.

There was a statistically significant association between Gram-negative bacterial growth in exit samples and hospital stay duration. This tallies with the findings of other studies that showed that staying longer in hospitals increases the likelihood of getting an infection [39–41]. The longer a patient is admitted the higher the likelihood of getting in contact with infectious pathogenic bacteria from the hospital premises, HCWs or other patients.

This study showed a significant association between visitors offering services like feeding, turning patients, changing patients’ clothes, and growth of Gram-negative bacteria in exit samples. This finding tallies with the knowledge that contact with patients with persistent and or resistant bacteria facilitates the spread of bacteria [42].

Lack of hand washing at entrance and exit was associated with visitors’ hand contamination. This finding is similar to that reported in several studies that have demonstrated that contaminated hands are couriers of infectious agents when not properly decontaminated [18,42,43]. However, there was an association between washing hands and having Gram-negative bacteria on hands at entrance which does not tally with scientific knowledge that hand washing reduces or removes bacterial contamination. This disparity could be due to not washing hands properly thus visitors retaining bacteria on their hands.

The isolation of multidrug-resistant (MDR) Gram-negative bacteria upon hospital entry and exit has serious implications. For hospitalized patients, it increases the risk of nosocomial infections, leading to higher treatment costs, prolonged hospital stays, and, in severe cases, death. For the community, the release of MDR bacteria from hospitals raises the risk of community spread. These study findings underscore the need to strengthen IPC implementation in the hospital setting to prevent spread of pathogenic and multidrug resistant bacteria that lead to multidrug infections that can result in adverse outcomes like increased morbidity and mortality.

### Study limitations

This research is subject to some limitations. Firstly, participants were swabbed at the ward entrance rather than at the hospital entrance, this implies that the contamination found on their hands may not have originated from the community, as some had already touched hospital surfaces. Secondly, since visitors’ hands were not sanitized after the initial swab at the ward entrance, the bacteria isolated at the exit cannot be definitively traced back to the hospital *Mitigation*: Prevalence obtained was not reported as coming from the hospital directly. All practices at the hospital were self-reported and thus could not be verified. *Mitigation*: All practices were reported as self-reported to avoid misleading.

## Conclusion and recommendations

There is a significantly high prevalence of bacterial contamination brought into and taken out of hospitals by visitors. This study underlines the critical role that visitors play in the spread of bacterial contamination, including MDR and ESBL-producing bacteria, within healthcare settings. The higher prevalence of ESBL-producing bacteria being taken out of hospitals by visitors highlights a significant public health concern, particularly for vulnerable populations such as immunocompromised patients. To mitigate the risk of spreading MDR infections, it is crucial to implement and reinforce strict infection control measures, including rigorous hand hygiene protocols and limitations on visitor exposure within hospitals

### To the regional referral hospitals

Set up hand washing facilities at the entrance/exit of wards and hospital buildings in order to facilitate easy access to hand washing facilities by the visitors and thus help prevent spread of bacterial infections. Establish and enforce a system of ensuring visitors comply with proper hand hygiene before and after entering the hospital wards similar to what was done during the COVID-19 pandemic. Visual/audio reminders can be placed near hand-washing facilities to remind visitors to comply with hand washing [44].

### To the Ministry of Health

Develop a policy that encourages compliance with hand hygiene practices by all visitors to patients admitted in public and private hospitals. Strengthen implementation of the National IPC guidelines in all hospitals. Strengthen the policies on antibiotic stewardship, antimicrobial surveillance, and infection control to help guide empirical antibiotic therapy and prevent the spread of MDR bacteria and antibiotic drug resistance.

### To the scientific community

Further research should be done, involving many healthcare facilities in both urban and rural settings with a focus on visitors’ contribution to the bacterial pool, especially MDR bacteria, and associated factors.

## Supporting information

S1 Table Antimicrobial susceptible pattern of Gram-negative bacteria in sample A and B contaminating hands of patients’ visitors. (DOCX)

S1 Data Anonymized. (CSV)
