# Supplementary material for: Prevalence and antimicrobial susceptibility pattern of gram-negative bacteria contaminating the hands of patients’ visitors at regional referral hospitals in Dar-es-Salaam
Source: PLoS One. 2025 Mar 26;20(3):e0320700. doi: 10.1371/journal.pone.0320700 (PMC11940574; doi:10.1371/journal.pone.0320700)
Supplement: S1 Table — (DOCX) [file pone.0320700.s001.docx]

**Table: Antimicrobial susceptibility test results indicating the sensitivity of recovered Gram-negative bacteria in sample A and B.**

Sample A

| **Sample A negative bacteria** |  | **AUG** | **AMP** | **FEP** | **CAZ** | **IMI** | **CN** | **AK** | **CIP** | **CTX** | **CRO** |
| --- | --- | --- | --- | --- | --- | --- | --- | --- | --- | --- | --- |
|  | Total |  |  |  |  |  |  |  |  |  |  |
| Acinetobacter Species | 13 | NA(NA) | NA(NA) | 8(61.5) | 9(69.2) | 12(92.3) | 12(92.3) | 13(100) | 13(100) | 2(15.4) | 4(30.8) |
| Enterobacter species | 12 | 2(16.7) | 1(8.3) | 10(83.3) | 7(58.3) | 12(100) | 10(83.3) | 10(83.3) | 10(83.3) | 5(41.7) | 10(83.3) |
| Klebsiella species | 37 | 12(32.4) | 0(0) | 23(62.2) | 20(54.1) | 33(89.2) | 31(83.8) | 29(78.4) | 17(45.9) | 11(29.7) | 24(64.9) |
| Pseudomonas species | 22 | NA(NA) | NA(NA) | 22(100) | 22(100) | 22(100) | 21(95.5) | 21(95.5) | 22(100) | NA(NA) | NA(NA) |
| Other bacteria | 7 | 3(42.9) | 0(0) | 7(100) | 6(85.7) | 7(100) | 7(100) | 7(100) | 7(100) | 4(57.1) | 7(100) |

NA- Not applicable

Sample B

| **Sample B negative bacteria** |  | **AUG** | **AMP** | **FEP** | **CAZ** | **IMI** | **CN** | **AK** | **CIP** | **CTX** | **CRO** |
| --- | --- | --- | --- | --- | --- | --- | --- | --- | --- | --- | --- |
|  | Total |  |  |  |  |  |  |  |  |  |  |
| Acinetobacter Species | 28 | NA(NA) | NA(NA) | 25(89.3) | 20(71.4) | 27(96.4) | 27(96.4) | 27(96.4) | 26(92.9) | 4(14.3) | 7(25) |
| Enterobacter species | 10 | 1(10) | 1(10) | 6(60) | 6(60) | 9(90) | 7(70) | 9(90) | 7(70) | 4(40) | 6(60) |
| Klebsiella species | 57 | 14(24.6) | 1(1.8) | 25(43.9) | 22(38.6) | 43(75.4) | 44(77.2) | 46(80.7) | 22(38.6) | 12(21.1) | 27(47.4) |
| Pseudomonas species | 26 | NA(NA) | NA(NA) | 26(100) | 26(100) | 26(100) | 26(100) | 26(100) | 26(100) | NA(NA) | NA(NA) |
| other bacteria | 12 | 6(50) | 2(16.7) | 11(91.7) | 10(83.3) | 11(91.7) | 11(91.7) | 10(83.3) | 8(66.7) | 6(50) | 10(83.3) |

NA- Not applicable
